# From Dyspnea to Suspected Breast Cancer: A Post-traumatic Hematoma as a Diagnostic Pitfall

**DOI:** 10.7759/cureus.114280

**Published:** 2026-08-10

**Authors:** Osnat Cohen, Nicolas Carême, Roanne Van Steen, Inge Verslegers, Ali Ramadhan, Filip Thiessen, Wiebren A Tjalma

**Affiliations:** 1 Multidisciplinary Breast Clinic, Department of Obstetrics and Gynaecology, Antwerp University Hospital, Edegem, BEL; 2 Faculty of Medicine and Health Sciences, University of Antwerp, Antwerp, BEL; 3 Multidisciplinary Breast Clinic, Department of Radiology, Antwerp University Hospital, Edegem, BEL; 4 Multidisciplinary Breast Clinic, Department of Plastic, Reconstructive and Aesthetic Surgery, Antwerp University Hospital, Edegem, BEL; 5 Multidisciplinary Breast Clinic, Department of Pathology, Antwerp University Hospital, Edegem, BEL; 6 Multidisciplinary Breast Clinic, Department of Obstetrics and Gynecology, Antwerp University Hospital, Edegem, BEL

**Keywords:** breast cancer mimic, breast hematoma, breast imaging reporting and data system (bi-rads), breast trauma, clinical case report, core needle biopsy, diagnostic pitfall, incidental breast lesion, multidisciplinary approach, multimodal imaging approach

## Abstract

Post-traumatic breast hematomas are uncommon but clinically relevant, as they may closely mimic breast cancer on both clinical examination and imaging. These hematomas may be large and persistent, particularly in patients receiving anticoagulant therapy. Conversely, breast cancer itself may present with hematoma formation, creating a diagnostic pitfall. With the increasing use of cross-sectional imaging for non-breast indications, incidental breast findings are detected with growing frequency. Although many of these findings are benign, their imaging appearance can occasionally mimic malignancy, often necessitating further diagnostic evaluation. We report the case of a 76-year-old woman who presented with progressive dyspnea, leading to a chest computed tomography (CT) for pulmonary evaluation. This examination revealed an incidental breast mass-like lesion. The patient reported a recent fall with subsequent ecchymosis of both breasts while on chronic anticoagulant therapy with apixaban. Clinical examination six months after the fall still demonstrated a large palpable mass measuring 60 x 50 mm with associated skin retraction in the right breast. The lesions in the left breast had resolved completely. Mammography, ultrasound, and magnetic resonance imaging (MRI) resulted in a Breast Imaging Reporting and Data System (BI-RADS) IV classification. The clinical examination in combination with the breast imaging was suggestive of locally advanced breast cancer. A core biopsy showed benign findings consistent with an organizing hematoma. It could, however, not rule out a malignant lesion. Following a multidisciplinary team meeting discussion, it was decided to repeat the clinical examination, imaging, and biopsy after two months. After two months, the deformation of the breast had increased, and the image was still BI-RADS IV, but the repeated core biopsy confirmed a complex organized hematoma without evidence of carcinoma. This case illustrates the diagnostic challenges associated with post-traumatic breast lesions. In this patient, clinical examination and imaging were unable to reliably distinguish an organized hematoma from breast cancer despite a clear history of trauma and anticoagulant therapy. Histological confirmation was therefore required. Persistent clinical-radiological discordance required a multidisciplinary approach. The decision to repeat the assessment after an appropriate follow-up was essential to ensure diagnostic safety and avoid both delayed cancer diagnosis and unnecessary overtreatment.

## Introduction

Dyspnea on exertion is a common presenting complaint in elderly patients and warrants a broad differential diagnosis, including cardiac, pulmonary, and oncological causes [1]. A computed tomography (CT) of the chest is therefore frequently performed to evaluate conditions, such as heart failure, pulmonary embolism, interstitial lung disease, or malignancy [2]. With the increasing use of chest CT for non-breast-related indications or positron emission tomography-CT (PET-CT) scans, incidental breast findings, so-called incidentalomas, are detected with increasing frequency [3-6].

At present, breast incidentalomas are detected in 0.1-7.6% of CT scans, 0.3-0.4% of PET-CT scans, and 0.01-9.3% of non-breast magnetic resonance imaging (MRI) examinations [5-7]. Although most incidental breast lesions are benign, approximately one in five is malignant in published series [5-7]. Consequently, any newly detected incidental breast lesion, particularly in postmenopausal women, should be considered malignant until proven otherwise. Referral to a recognised breast clinic for triple assessment (i.e., clinical examination, imaging and biopsy when indicated) is therefore recommended. The growing detection of incidental breast findings represents both an opportunity for early cancer diagnosis and a clinical challenge, as it may also lead to patient anxiety, overdiagnosis, and unnecessary interventions if not managed in a structured manner [5,6].

Post-traumatic breast hematomas represent a benign yet clinically challenging entity within this context. Depending on their size, chronicity, and degree of organization, hematomas may present as palpable masses with skin retraction and architectural distortion on imaging [8]. Fibrosis, fat necrosis, and chronic inflammation contribute to these suspicious clinical and imaging appearances. These features closely overlap with those of invasive breast cancer or recurrent breast cancer [9]. The diagnostic challenge is further amplified in elderly patients and in those receiving anticoagulant therapy, in whom hematomas are often larger, more persistent, and less likely to resolve spontaneously.

Importantly, the diagnostic dilemma is bidirectional. While breast hematomas may mimic malignancy, breast cancer itself can occasionally present with hematoma formation, thereby masking an underlying carcinoma [10,11]. Reliance on clinical history and imaging may therefore result in both overdiagnosis and delayed cancer detection.

We present a case in which the evaluation of dyspnea on exertion led to a chest CT with an incidental breast finding. In the setting of a large palpable breast mass, skin retraction, and suspicious imaging features, a diagnosis of locally advanced breast carcinoma was initially considered. This case highlights the diagnostic complexity of post-traumatic breast lesions, the limitations of imaging in differentiating benign from malignant disease, and the need for a structured, multidisciplinary diagnostic approach. The aim of this report is to illustrate how a post-traumatic breast hematoma can closely mimic breast cancer, to emphasize the importance of clinical-radiological correlation with repeated histological assessment, and to raise awareness of diagnostic pitfalls related to incidental breast findings on cross-sectional imaging.

## Case presentation

A 76-year-old woman was referred for further evaluation in the context of progressive dyspnea on exertion. Her medical history was significant for heart failure with preserved ejection fraction (HFpEF), morbid obesity (body mass index 38.4 kg/m²), recurrent atrial fibrillation treated with antiarrhythmic therapy, and moderate obstructive sleep apnea, for which continuous positive airway pressure therapy had been discontinued because of poor tolerance. She was on chronic anticoagulation therapy with Apixaban. There was no personal or familial history of breast cancer.

The patient reported exertional dyspnea, with clear worsening over the preceding six months, for which she was followed by cardiology and pulmonology. Pulmonary function testing demonstrated a mild restrictive ventilatory defect (total lung capacity (TLC) 3.75 L, 76% predicted) with reduced forced vital capacity (FVC) (78% predicted) and preserved forced expiratory volume in one second/FVC ratio (72%). Diffusing capacity was normal (diffusing capacity of the lungs for carbon monoxide (DLCO) 80% predicted), indicating preserved gas transfer. Review of serial pulmonary function tests performed between 2016 and 2021 demonstrated a stable restrictive pattern without significant decline in lung function over time. Given the patient's recent worsening of symptoms despite previously stable pulmonary function, further evaluation with thoracic CT was undertaken.

Her previous history included a fall six months earlier, during which she struck her chest against the edge of a bathtub. This resulted in extensive bilateral breast hematomas, requiring short-term hospitalization. While the hematoma of the left breast resolved completely, a persistent deformity and palpable mass remained in the right breast, which did not regress over time.

**Figure 1:**
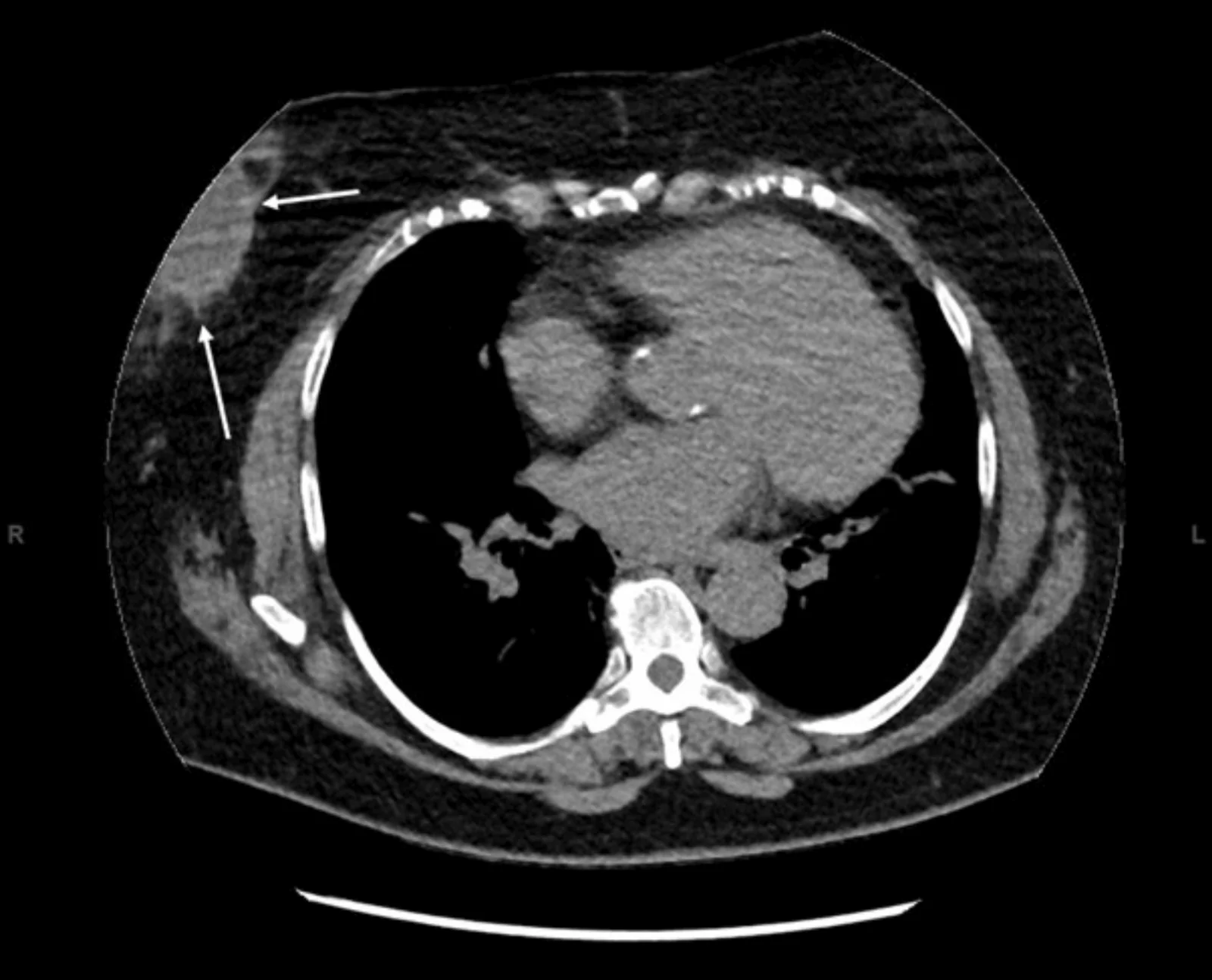
Axial CT image of the chest performed for the evaluation of dyspnea, demonstrating an incidental high-density mass-like lesion (arrows) in the partially imaged right breast.

**Figure 2:**
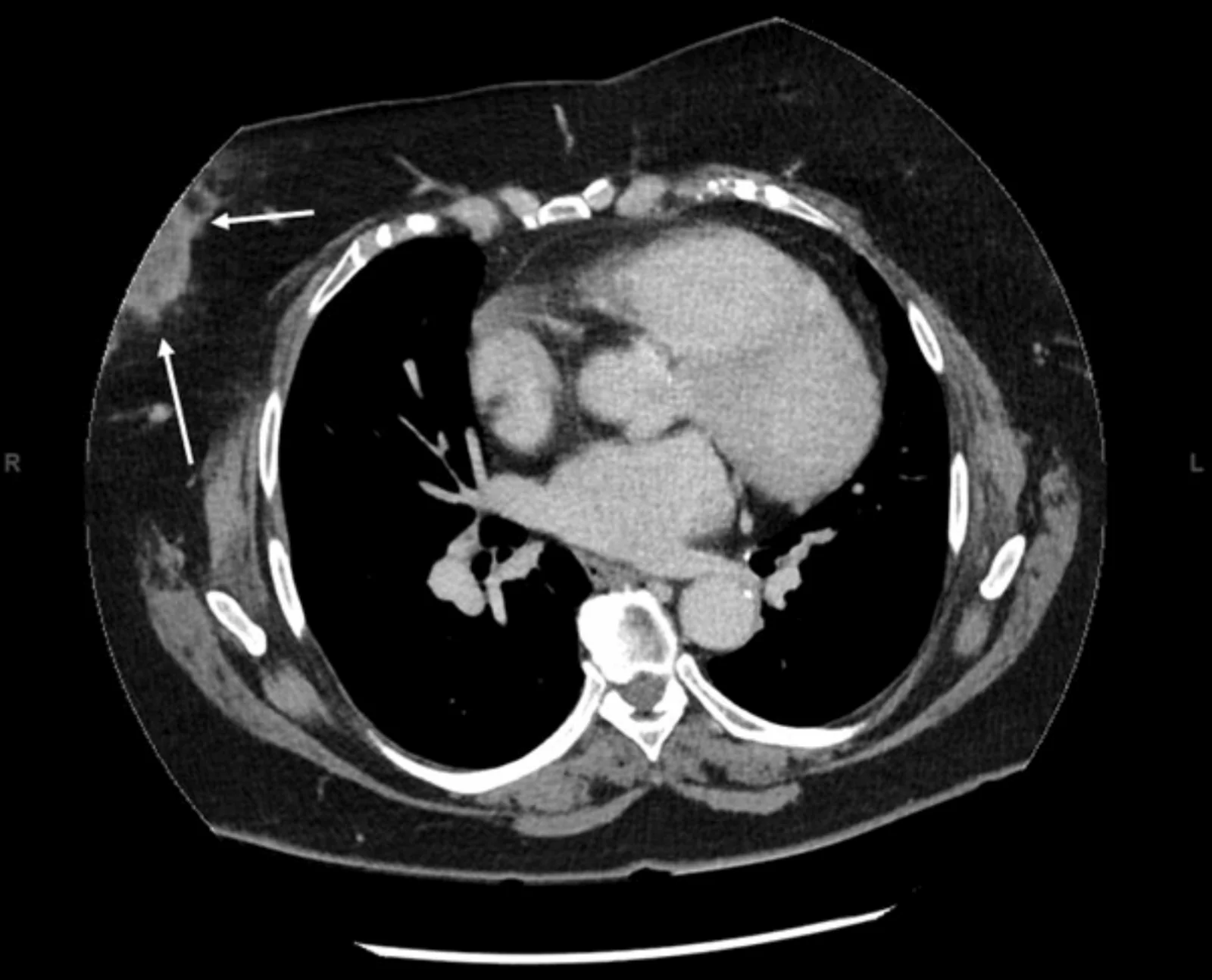
Follow-up axial chest CT performed six months later showed persistent abnormalities in the right breast (arrows), with resolution of the pulmonary findings.

The initial chest CT for the evaluation of dyspnea demonstrated pulmonary findings compatible with bronchial disease and focal bronchiectasis. An incidental high-density mass-like lesion was noted in the partially imaged right breast, prompting further dedicated breast imaging (Figure 1). The follow-up chest CT, performed six months later, showed resolution of the transient pulmonary abnormalities, while the focal bronchiectasis remained unchanged. The previously identified right breast lesion remained visible (Figure 2).

**Figure 3:**
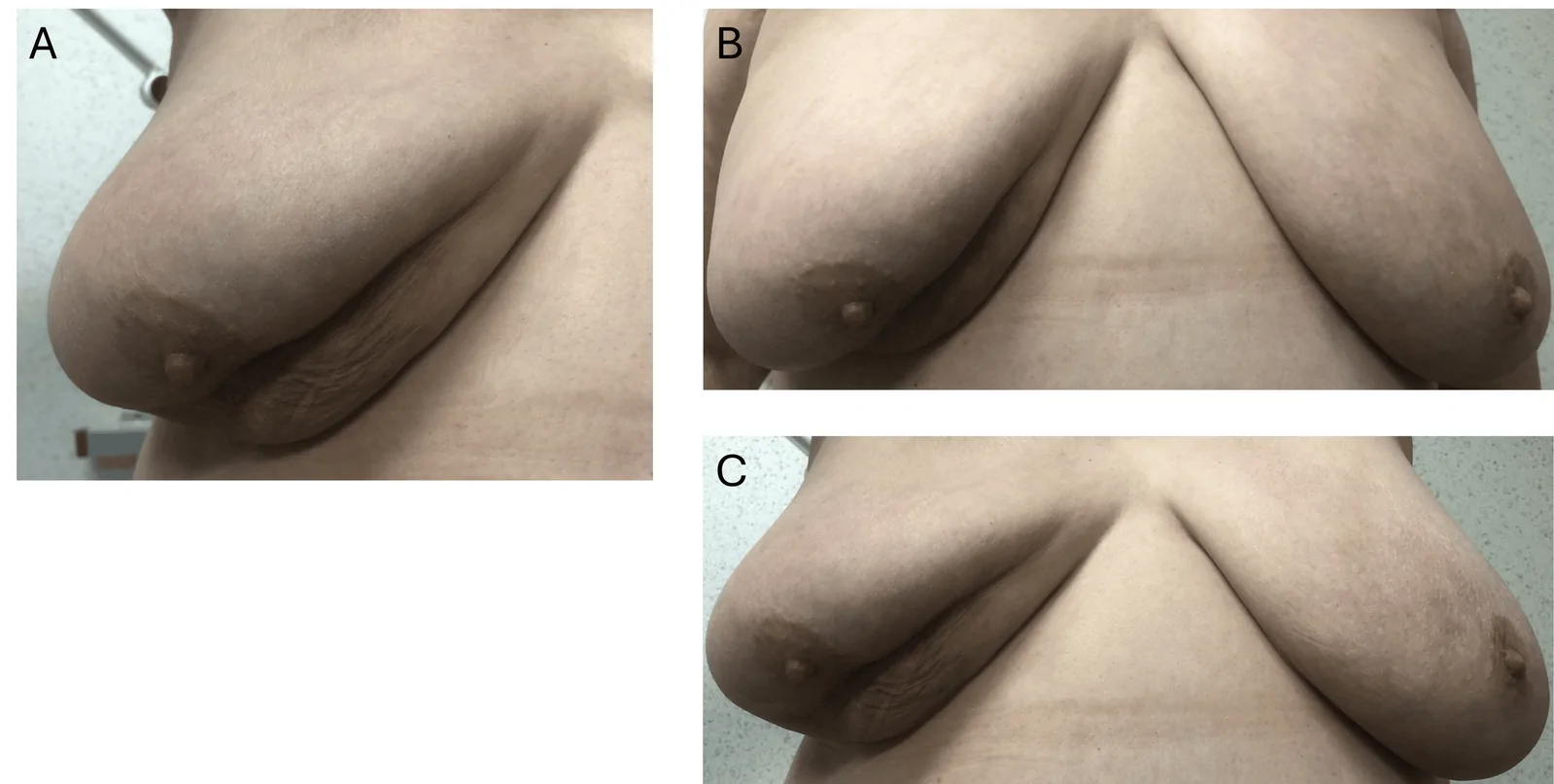
Clinical appearance of the right breast showing inferomedial skin retraction and residual ecchymosis six months after the traumatic fall. (A) Detail of the right breast; (B) arms next to the body; (C) arms stretched out.

She denied breast pain, nipple discharge, weight loss, or other systemic symptoms. Clinical examination, six months after the fall, revealed a symmetrical and unremarkable left breast. Examination of the right breast showed a firm palpable mass measuring approximately 6 cm in the inferomedial quadrant, associated with skin retraction and residual ecchymosis and discoloration of the lower half of the breast (Figure 3A-3C). The mass appeared adherent to the skin but not to the underlying muscle. No nipple retraction was present. No axillary or supraclavicular lymphadenopathy was detected bilaterally.

**Figure 4:**
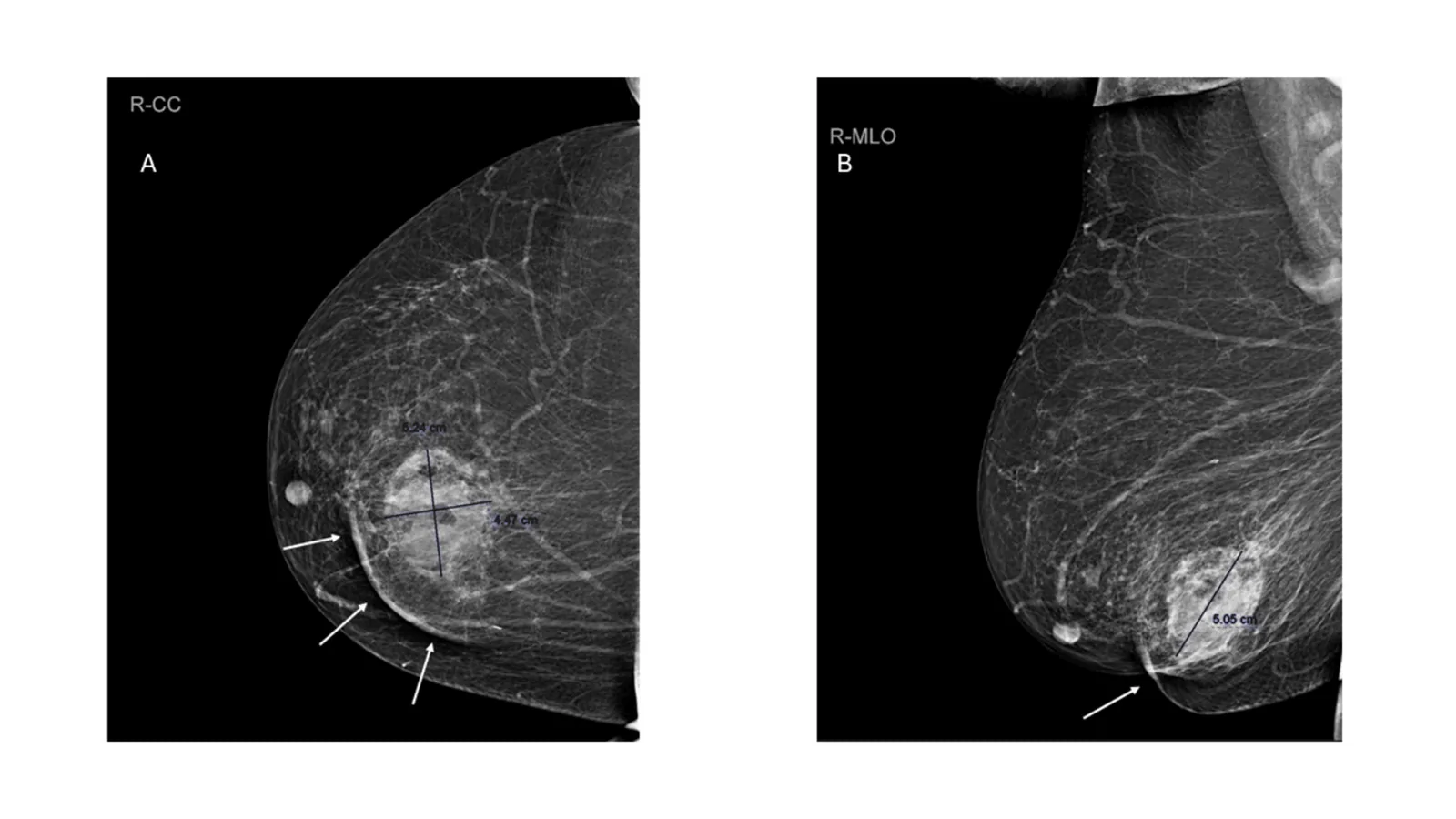
Mammography of the right breast showing an ill-defined mass measuring 5.2 × 4.5 × 5.0 cm (measurements, A–B) and skin retraction (arrows, A–B).

**Figure 5:**
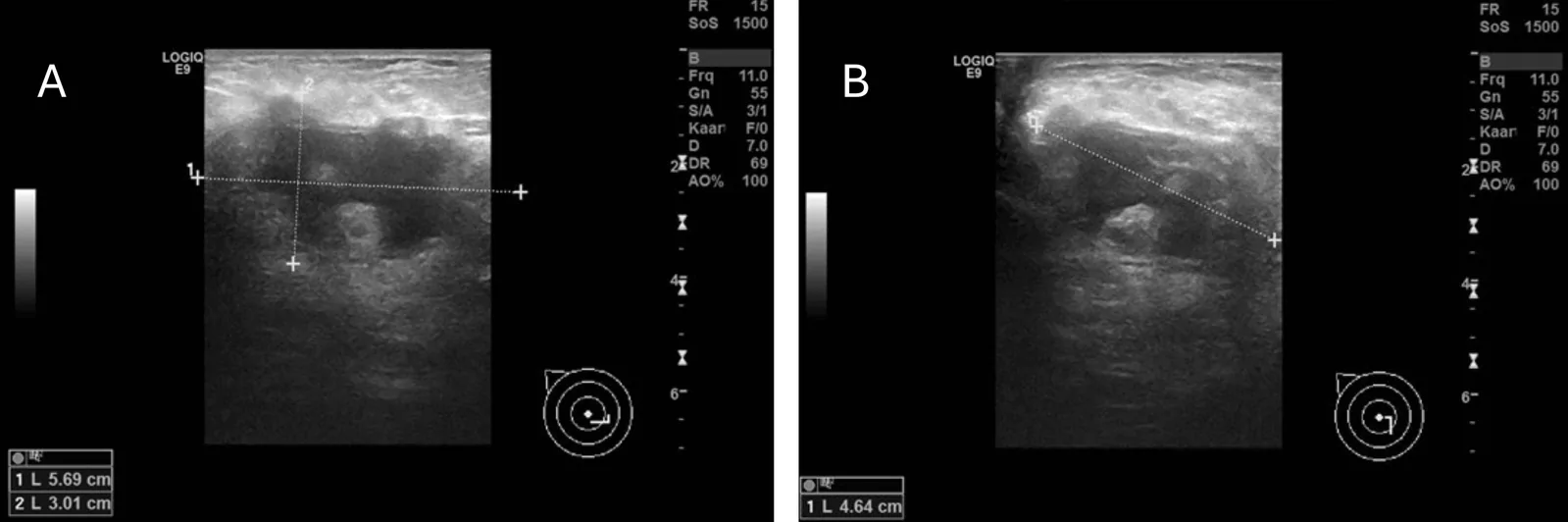
Ultrasound of the right breast revealing an ill-defined heterogeneous mass measuring 5.0 × 4.6 × 3.0 cm with posterior acoustic enhancement (A–B) (BI-RADS IV).

**Figure 6:**
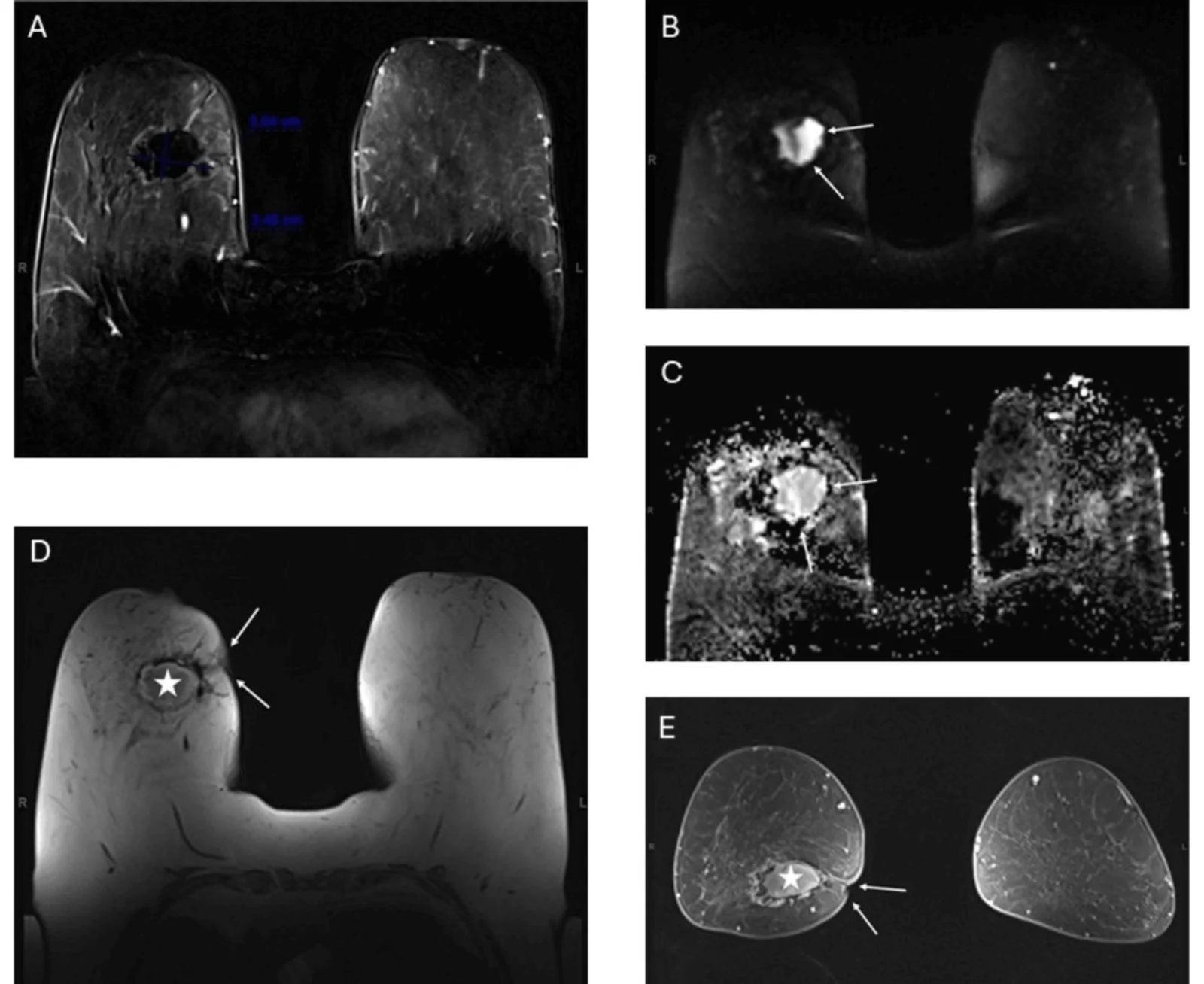
Breast magnetic resonance imaging showing a multicompartmental T1-hyperintense mass (stars, D–E) measuring 4.6 × 3.5 × 4.9 cm in the inferomedial right breast with subtle peripheral enhancement (measurements, A), no diffusion restriction (arrows, B–C), and associated skin retraction (arrows, D–E) (BI-RADS IV).

Bilateral mammography and breast ultrasound performed revealed an ill-defined, inhomogeneous mass with skin retraction in the inferomedial quadrant of the right breast, measuring approximately 6 × 5 × 3 cm. The lesion showed mixed echogenicity without definite internal vascularization. Axillary lymph nodes appeared morphologically benign. The findings were classified as BI-RADS IV (Figures 4-5). Subsequent breast magnetic resonance imaging (MRI) demonstrated a multicompartmental mass infero-medially in the right breast, containing fluid and fat components, with associated skin retraction. The lesion showed predominantly peripheral enhancement and no diffusion restriction. Time-intensity curves revealed a gradual continuous enhancement pattern (type 1 curve). The estimated size ranged from 6.0 × 4.0 cm to 6.0 × 3.5 cm (Figure 6). The examination was classified as Breast Imaging Reporting and Data System (BI-RADS) IV. Unfortunately, the MRI could not reliably differentiate between a regressing hematoma and a mass of another origin. The examination was classified as BI-RADS IV, and a biopsy was advised.

**Figure 7:**
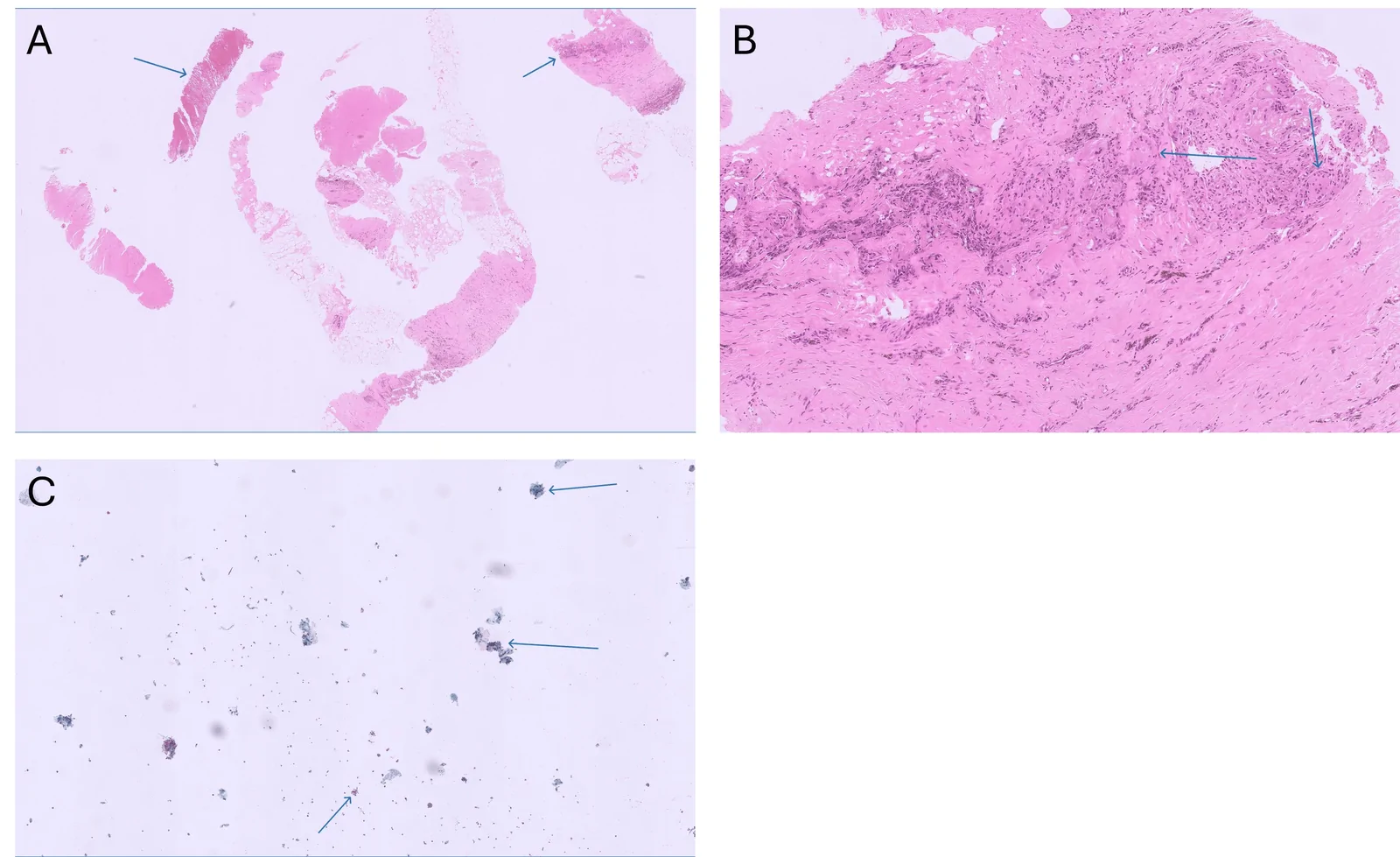
Core biopsy of an ill-defined mass in the inferomedial quadrant of the right breast. 7A: Core biopsy showing blood, fibrin, and granulation tissue with no epithelial component present, low-power view. Hematoxylin and eosin (H&E) ×1. 7B: Core biopsy showing granulation tissue and giant cell formation, medium-power view. H&E ×10. 7C: Cytology revealed blood and giant cells without suspicious epithelial cells.

Fine-needle aspiration cytology yielded blood without malignant cells. Ultrasound-guided core biopsy demonstrated granulation tissue, hemosiderin deposition, and multinucleated giant cells, consistent with an organizing post-traumatic hematoma, without evidence of atypia or malignancy (European Society of Breast Cancer Specialists (EUSOMA) C1/B1) (Figure 7A-7C). Based on the presence of a large persistent palpable breast mass, associated skin retraction, and BI-RADS IV findings on mammography, ultrasound, and MRI, the initial differential diagnosis included locally advanced breast cancer (clinical T3N0) versus a chronic post-traumatic breast hematoma. The performed core biopsy could neither confirm nor rule out a malignancy. Following a multidisciplinary team meeting discussion, it was decided to repeat the clinical examination, imaging, and biopsy after two months.

**Figure 8:**
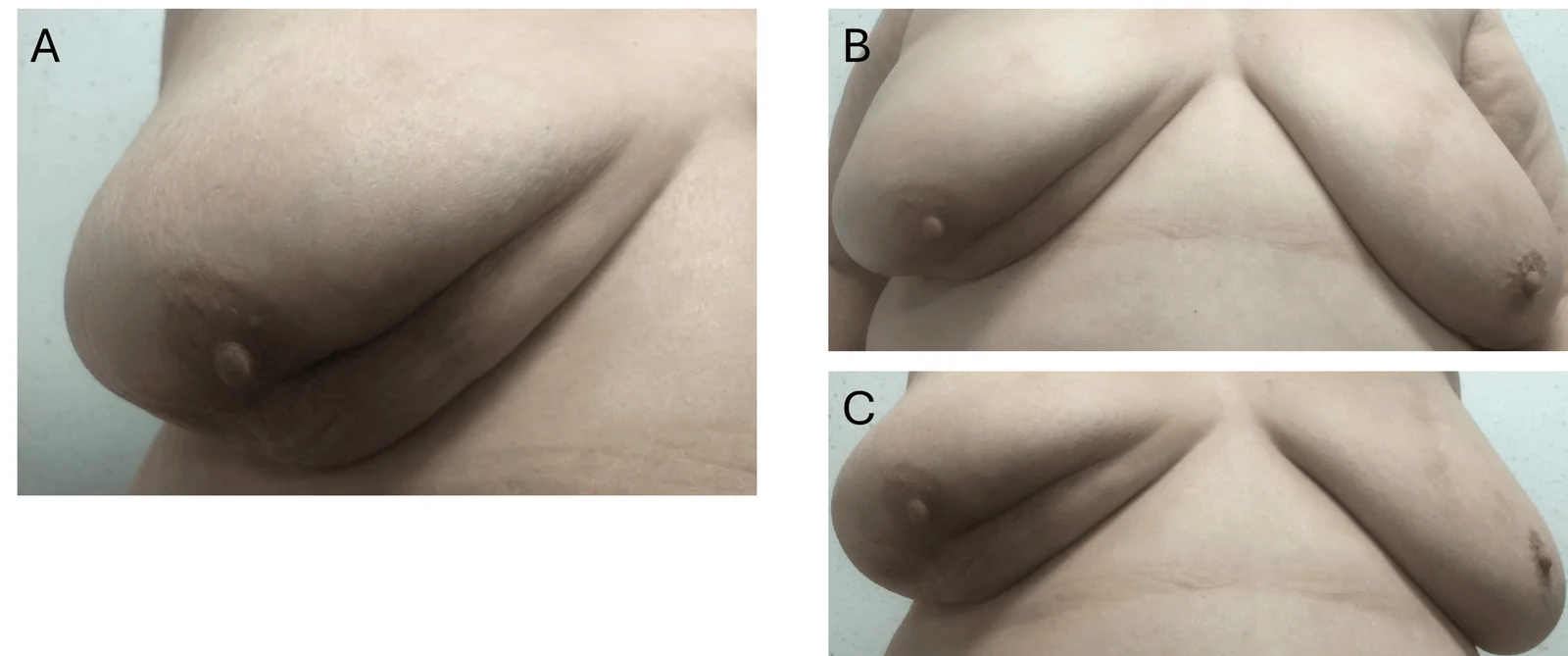
Clinical appearance of the right breast showing inferomedial skin retraction and residual ecchymosis eight months after the traumatic fall. (A) Detail of the right breast; (B) arms next to the body; (C) arms stretched out.

Clinical examination, two months later (eight months after the fall), revealed that the skin retraction in the right breast had further increased (Figure 8A-8C).

**Figure 9:**
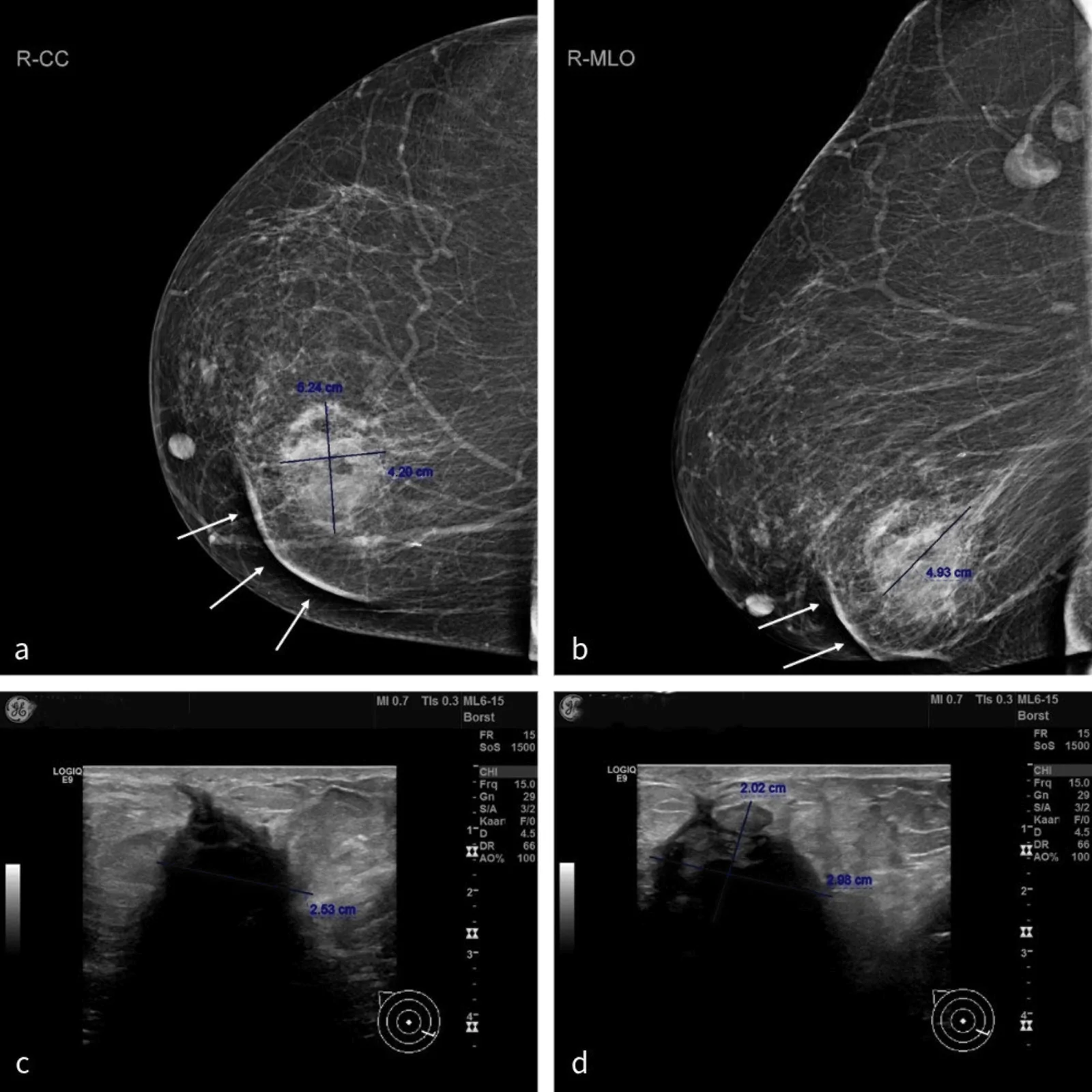
Mammography performed two months later showed an unchanged ill-defined mass measuring 6.2 × 4.2 × 4.9 cm in the inferomedial right breast (measurements, A–B) with persistent skin retraction (arrows, A–B). Ultrasound of the right breast revealed an ill-defined heterogeneous mass with posterior acoustic shadowing (C–D) (BI-RADS IV). BI-RADS: Breast Imaging Reporting and Data System

**Figure 10:**
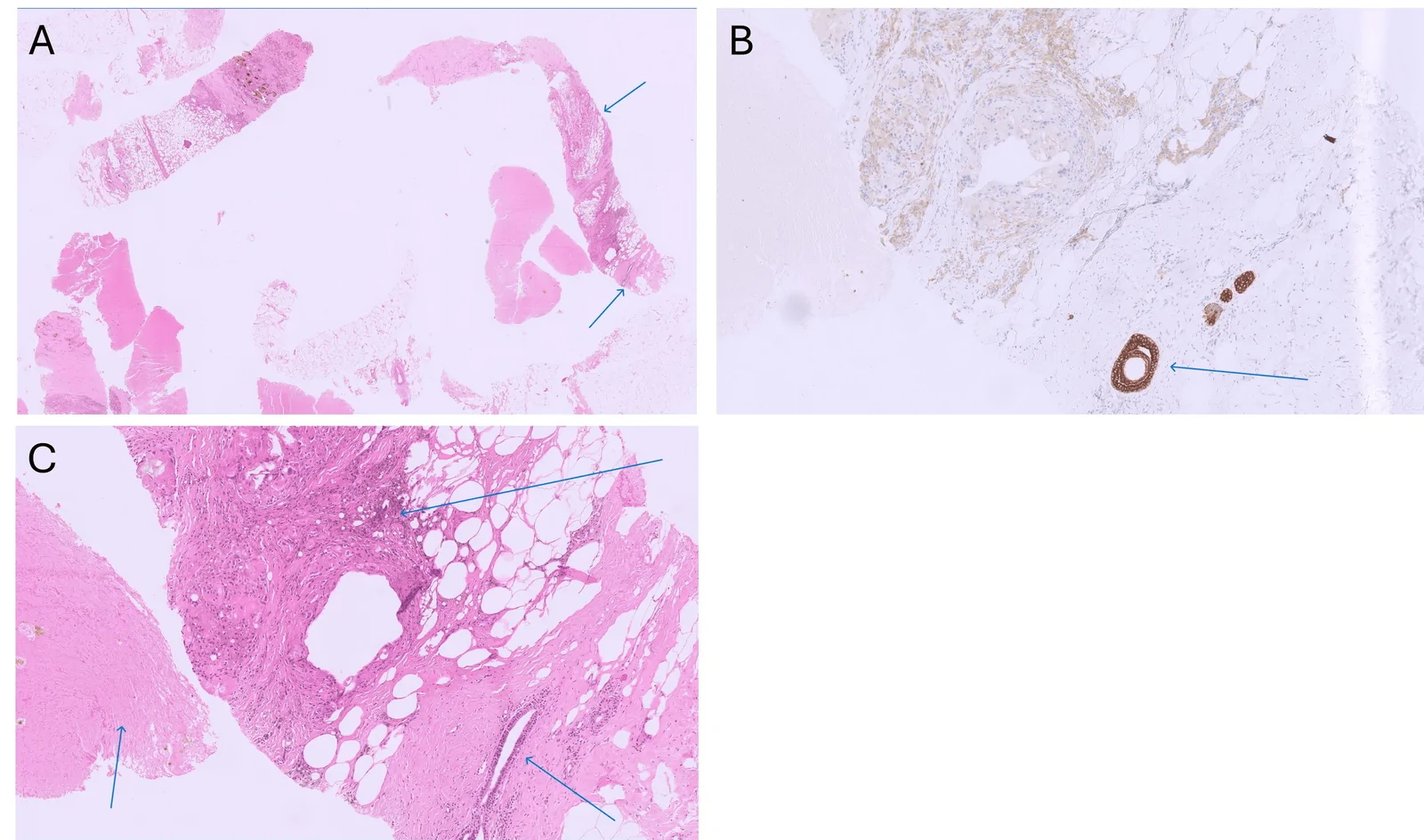
Core biopsy of an ill-defined mass in the inferomedial quadrant of the right breast, performed two months after the first biopsy and eight months after the fall. 10A: Ultrasound-guided core biopsy showing organizing hematoma, hemosiderin deposition, and residual normal breast epithelium without evidence of malignancy, low-power view. Hematoxylin and eosin (H&E) ×1. 10B: Immunohistochemical stain for pan-cytokeratin highlighting residual normal breast epithelium without evidence of malignancy, medium-power view. H&E ×10. 10C: Ultrasound-guided core biopsy showing organizing hematoma with fat necrosis and hemosiderin deposition, with residual benign-looking ducts present, medium-power view. H&E ×10.

The repeated mammography and ultrasound confirmed the increased retraction and a slightly progressive lesion, which was again classified as BI-RADS IV (Figure 9). The repeated core biopsy revealed progressive organization of the hematoma with prominent fat necrosis, dense fibrosis, and extensive hemosiderin deposition (Figure 10A-10C). To exclude occult carcinoma within the organizing hematoma, a recognized diagnostic pitfall, ancillary immunohistochemical staining for pancytokeratin was performed (Figure 10B), which highlighted only pre-existing occasional benign ductal structures without evidence of invasive carcinoma. The final pathological diagnosis was a benign, complex organized hematoma (EUSOMA B1). The patient was reassured regarding the benign nature of the breast deformity. She was furthermore informed about the possibility of corrective surgery by a plastic surgeon.

## Discussion

Breast hematoma formation is common and typically multifactorial. They are often associated with blunt trauma, anticoagulant or antiplatelet therapy, iatrogenic injury, and advanced age (Table 1) [4,8-10,12-15].

**Table 1:** Risk factors for breast hematoma formation and diagnostic implications

Risk factor	Underlying mechanism	Typical clinical context	Impact on imaging and diagnosis
Blunt breast trauma	Rupture of intramammary vessels with blood extravasation	Falls, direct chest impact, motor vehicle accidents	Acute or chronic mass, skin retraction, architectural distortion mimicking carcinoma
Anticoagulant therapy	Impaired hemostasis leading to larger and persistent hemorrhage	DOACs (apixaban), VKA, aspirin, dual antiplatelet therapy	Large, slowly resolving hematomas; higher risk of organization and malignancy mimicry
Advanced age	Increased tissue fragility and reduced resorption capacity	Elderly patients, postmenopausal women	Higher likelihood of persistent masses and diagnostic uncertainty
Iatrogenic injury	Vessel damage during intervention	Core biopsy, vacuum biopsy, breast surgery, interventional radiology	Post-procedural mass with suspicious imaging features
Chronic hematoma organization	Fibrosis, fat necrosis, hemosiderin deposition	Long-standing or untreated hematomas	Irregular margins, spiculation, enhancement and diffusion restriction on MRI
Fat necrosis	Adipocyte destruction following trauma or surgery	Trauma, surgery, radiotherapy	Spiculated mass, calcifications, enhancement mimicking malignancy
Inflammatory response	Granulation tissue and giant cell reaction	Organizing hematomas	Persistent enhancement on MRI, false-positive BI-RADS IV/V
Underlying malignancy	Tumor-related hemorrhage	Breast cancer presenting with bleeding	Hematoma masking carcinoma or delaying diagnosis

While most acute or subacute breast hematomas resolve spontaneously, larger lesions or those undergoing chronic organization may persist and evolve into complex masses [16]. During this process, hemorrhagic content is gradually resorbed and replaced by fibrosis and fat necrosis development, leading to permanent structural distortion of the breast [9,13,16]. In this chronic phase, typical clinical signs, such as skin bruising, are often absent, and a clear history of trauma may no longer be recalled, particularly in elderly patients or those receiving anticoagulant therapy [4,10]. This biological evolution explains the wide spectrum of misleading imaging appearances. Organized hematomas may present as ill-defined or spiculated masses with skin retraction on mammography and ultrasound, increased stiffness on elastography, persistent or peripheral enhancement on MRI, and even increased FDG uptake on PET-CT due to ongoing inflammatory activity [16,17]. These features closely overlap with those of invasive breast cancer and substantially limit the specificity of imaging. Importantly, the diagnostic challenge is bidirectional. While benign hematomas may mimic malignancy, breast cancer itself may present with hemorrhage or blood-filled collections, either due to tumor necrosis or erosion of intratumoral vessels [4,10]. Consequently, imaging alone is insufficient to reliably distinguish chronic hematoma from malignancy. In the absence of unequivocal benign features, histological confirmation and structured follow-up remain mandatory. 

From a clinical perspective, the presence of a bleeding risk factor and a recent history of trauma may intuitively suggest a benign etiology. In selected cases, a conservative approach with clinical follow-up is therefore justified. Small hematomas may resolve within several months, as observed in the contralateral left breast of our patient. However, this strategy becomes problematic when hematomas persist, enlarge, or progressively organize over time. In such circumstances, delayed assessment may lead to increasing skin retraction and breast deformation, as seen in the present case in the right breast. Imaging may provide additional information but can also increase diagnostic uncertainty. The characteristics of the organized hematomas may closely mimic features of breast cancer or obscure an underlying malignancy, placing them at the intersection of incidental breast findings and oncologic diagnostic pitfalls [10,13].

In the present case, the post-traumatic breast hematoma in the right breast was first highlighted as a breast abnormality on chest CT performed for non-breast-related indications, in a patient who also had clinically evident breast deformation. Incidental breast lesions detected on cross-sectional imaging are relatively uncommon but clinically significant, particularly in older women. Their relevance is driven by the global burden of breast cancer, which remains the most frequently diagnosed malignancy in women worldwide, accounting for approximately 2.3 million new cases annually and continuing as a leading cause of cancer-related mortality [18]. A large retrospective cohort study reported a malignancy rate of 21% among incidental breast findings with available follow-up, and advanced age was identified as an independent predictor of cancer [7]. Reported malignancy rates differ between imaging modalities, ranging from approximately 8% for MRI-detected lesions to 35% for chest CT and 55% for PET-CT. These rates are based on different studies and are therefore not directly comparable. Consequently, any newly detected incidental breast lesion, particularly in postmenopausal women, should be considered as potentially malignant until proven otherwise. Against this background of increasing incidental breast findings and substantial malignancy risk, post-traumatic breast hematoma emerges as a key diagnostic pitfall, particularly when there is no clinical context or when trauma is not actively reported or occurred months earlier.

This report is limited by its single-case nature, which restricts generalizability. Moreover, diagnostic uncertainty remains inherent to post-traumatic breast lesions, as imaging may be inconclusive and biopsy subject to sampling error. These limitations underscore the need for cautious interpretation and structured follow-up.

Post-traumatic breast hematoma as a diagnostic pitfall

Post-traumatic breast hematoma is a benign condition but represents a well-recognized diagnostic pitfall, particularly in older patients [19]. Traumatic breast injuries may lead to a wide spectrum of clinical and imaging findings that closely mimic breast cancer, particularly when trauma is followed by persistent structural changes within the breast parenchyma, as illustrated in our case [13,20]. With the increasing use of cross-sectional imaging for non-breast-related indications, incidental breast findings are detected more frequently, which triggers an extensive oncological work-up [3-5]. In this context, post-traumatic breast hematoma represents a classic diagnostic dilemma. It may closely simulate breast cancer, but it may also conceal an underlying malignancy [10,11,21]. An additional source of diagnostic confusion is the coincidental coexistence of traumatic breast lesions and breast cancer [21]. Moreover, late hematomas after breast reconstructive surgery with implants can mimic anaplastic large cell lymphoma [22-24].

Trauma-related breast hematomas result from rupture of intramammary vessels with subsequent extravasation of blood into the breast tissue. This bleeding may occur after direct blunt trauma, surgical procedures, or minimally invasive interventions, but can also follow relatively minor injury, particularly in vulnerable patients [10,19,25]. In elderly women, tissue fragility and reduced vascular elasticity increase susceptibility to bleeding, especially while using anticoagulant therapy. The combination can lead to extensive and persistent hematoma formation [4,12]. Seat belt injury, regardless of age, can also lead to active bleeding in the breast with the formation of a large hematoma [25]. Over time, hematomas may organize and evolve into complex lesions characterized by fibrosis, fat necrosis, and hemosiderin deposition, progressively losing their typical benign appearance. These chronic changes are the basis of the persistent clinical and radiological abnormalities that make post-traumatic breast hematomas such a challenging mimic of malignancy.

Limitations of imaging in post-traumatic breast lesions

The diagnostic uncertainty associated with post-traumatic breast hematomas is further compounded by the limitations of imaging. Mammography and ultrasound frequently demonstrate irregular margins, architectural distortion, and skin retraction, features that are indistinguishable from invasive breast cancer [10,13]. As a result, these first-line imaging modalities often fail to confidently differentiate benign post-traumatic changes from malignancy.

Magnetic resonance imaging (MRI) is therefore commonly used as a problem-solving modality. However, its diagnostic performance is also limited in the context of post-traumatic and inflammatory breast lesions. Organized or chronic hematomas may show persistent or peripheral contrast enhancement and diffusion restriction, closely overlapping with malignant MRI patterns [9,16]. Importantly, several benign breast entities frequently associated with trauma-related changes, such as fat necrosis, granulomatous mastitis, and lymphocytic mastopathy, may demonstrate aggressive enhancement kinetics on contrast-enhanced MRI, further limiting specificity [15]. Consequently, malignancy cannot be reliably excluded based on imaging alone, even when advanced techniques are applied. Similar diagnostic limitations have been described for contrast-enhanced mammography, where benign post-traumatic and inflammatory lesions may result in false-positive findings and trigger unnecessary oncological work-up [26,27].

Hematoma and breast cancer: a bidirectional diagnostic challenge

The present case exemplifies the inherent bidirectional diagnostic challenge between breast hematoma and breast cancer. Despite a documented history of breast trauma and anticoagulant use, the multimodal imaging classified the large persistent palpable mass with skin retraction as BI-RADS IV. The concern that this could be a locally advanced breast cancer was fully justified.

This concern is warranted because breast cancer itself may initially present with hemorrhage or hematoma formation, either due to tumor necrosis or erosion of intratumoral vessels, thereby mimicking benign post-traumatic changes or masking an underlying malignancy [8,11]. Several case reports have described invasive breast carcinomas that were initially interpreted as hematomas, underscoring the risk of delayed diagnosis when reassurance is given too early [28]. Conversely, benign post-traumatic hematomas, particularly when large, organized, or persistent, may closely mimic malignancy on clinical examination and imaging, leading to aggressive diagnostic work-up and potential overtreatment. This bidirectional overlap places clinicians at risk of both underdiagnosis and overdiagnosis. A presumed benign hematoma may delay cancer detection, while an overly aggressive approach may expose patients to unnecessary invasive procedures. Therefore, neither a history of trauma nor anticoagulant use should lead to premature diagnostic closure. Persistent clinical-radiological discordance mandates histological confirmation and structured follow-up. This balanced approach is essential to safely navigate the narrow margin between missing a malignancy and subjecting patients to unnecessary interventions. Given this bidirectional diagnostic uncertainty, imaging findings alone are insufficient to establish or exclude malignancy, and histological confirmation becomes pivotal.

Role of repeated histology and follow-up

When clinical suspicion persists, histological confirmation is mandatory. Imaging alone cannot reliably distinguish organized hematoma from malignancy. A single benign biopsy result does not reliably exclude malignancy, particularly when clinical and radiological findings remain discordant. The decision to repeat the biopsy after a two-month interval was based on the expected biological evolution of breast hematomas. During this period, hematomas typically undergo organization with resorption of blood products and the development of fibrosis and fat necrosis. The latter allows better correlation between imaging and histopathology. This interval reduces the risk of false-negative sampling in the early phase and facilitates a more reliable exclusion of malignancy [13,16].

Organized hematomas may yield benign histology while simultaneously masking an underlying carcinoma. This diagnostic limitation has been well described and underscores the risk of false reassurance based on a single tissue sample [4,9]. In such situations, repeated tissue sampling combined with structured clinical and imaging follow-up is essential to safely exclude malignancy. Recent case series and reviews emphasize that persistence or progression of suspicious features despite an initial benign biopsy should prompt reassessment rather than observation alone [19,20]. In the present case, malignancy could only be confidently ruled out after repeated core biopsies consistently demonstrated features of an organizing hematoma without evidence of carcinoma. Following the final benign diagnosis, no further breast-specific follow-up was considered necessary. At the time of writing, five years later, the patient remains well with no evidence of breast cancer.

Clinical implications and broader differential diagnosis

From a broader perspective, this case underscores the importance of a structured diagnostic strategy for traumatic breast lesions. A detailed trauma history should always be actively sought; however, its presence should never prematurely exclude malignancy. This is particularly relevant when breast abnormalities are detected incidentally. Incidental breast findings on chest CT, even when identified outside a dedicated breast imaging setting, require targeted evaluation with mammography and ultrasound, supplemented by MRI when indicated and, if necessary, followed by second-look ultrasound [19,29,30].

Diagnostic uncertainty is not limited to hematomas. Several benign breast lesions may closely mimic cancer on clinical examination and imaging. These entities are summarized in Table 2, illustrating that breast cancer mimics are common, diverse, and clinically relevant [4,8-10,12-15,17,20,22,23,26,27,31-42]. Awareness of these conditions is therefore essential for clinicians, radiologists, and pathologists.

**Table 2:** Benign breast lesions mimicking breast cancer and recommended diagnostic approach. *Elemental analysis refers to specialized techniques used to identify silicone material in tissue or fluid samples when routine histology is inconclusive. Common laboratory techniques include energy-dispersive X-ray spectroscopy (EDX/EDS), Fourier-transform infrared spectroscopy (FTIR), and scanning electron microscopy (SEM) combined with elemental mapping. These methods can demonstrate silicon as a chemical element, confirming that the lesion represents a silicone-induced inflammatory reaction rather than malignancy. ° Breast implant-associated anaplastic large cell lymphoma (BIA-ALCL) is an extremely rare form of non-Hodgkin lymphoma.

Lesion	Clinical and imaging features mimicking cancer	Diagnostic approach
Post-traumatic or spontaneous hematoma	Palpable mass, skin retraction, spiculated or ill-defined margins, BI-RADS IV/V, diffusion restriction; may mask occult carcinoma	Trauma/anticoagulant history; core biopsy, repeat core biopsy if discordant direct of after two to three months follow-up
Fat necrosis	Spiculated mass, architectural distortion, suspicious calcifications	Core biopsy +/-MRI
Radial scar / complex sclerosing lesion	Stellate or spiculated lesion	Vacuum-assisted biopsy or excision
(Idiopathic) Granulomatous mastitis	Firm mass with skin changes, inflammatory features	Core biopsy, microbiologic and histologic correlation
Breast abscess / mastitis	Irregular mass, skin thickening, trabecular distortion	Ultrasound +/- aspiration; biopsy if atypical
Fibroadenoma (atypical/infarcted)	Irregular margins, heterogeneous appearance, BI-RADS IV	Core biopsy +/- excision
Diabetic mastopathy (lymphocytic mastitis)	Hard painless mass, marked hypo-echogenicity	Core biopsy, avoid repeated surgery
Silicone granuloma / silicone mastopathy	Spiculated mass, calcifications, lymphadenopathy, abnormal uptake	Clinical history (implants), biopsy, elemental analysis*
Implant-related late hematoma	Rapid enlargement, peri-implant fluid/mass	Aspiration, cytology, biopsy; exclude BIA-ALCL°
Granular cell tumor	Spiculated hypoechoic mass	Core biopsy +/- excision
Diffuse dermal angiomatosis	Skin changes, trabecular thickening	Punch biopsy
Fibrocystic breast changes (focal)	Discrete mass or density without typical benign patterns	Core biopsy if suspicious
Pseudoangiomatous stromal hyperplasia (PASH)	Mass with heterogeneous echotexture	Core biopsy
Hamartoma	Heterogeneous mass with variable density	Core biopsy / excision if suspicious
Tubular adenoma	Solid mass sometimes spiculated	Core biopsy / excision

Importance of a structured multidisciplinary approach

**Figure 11:**
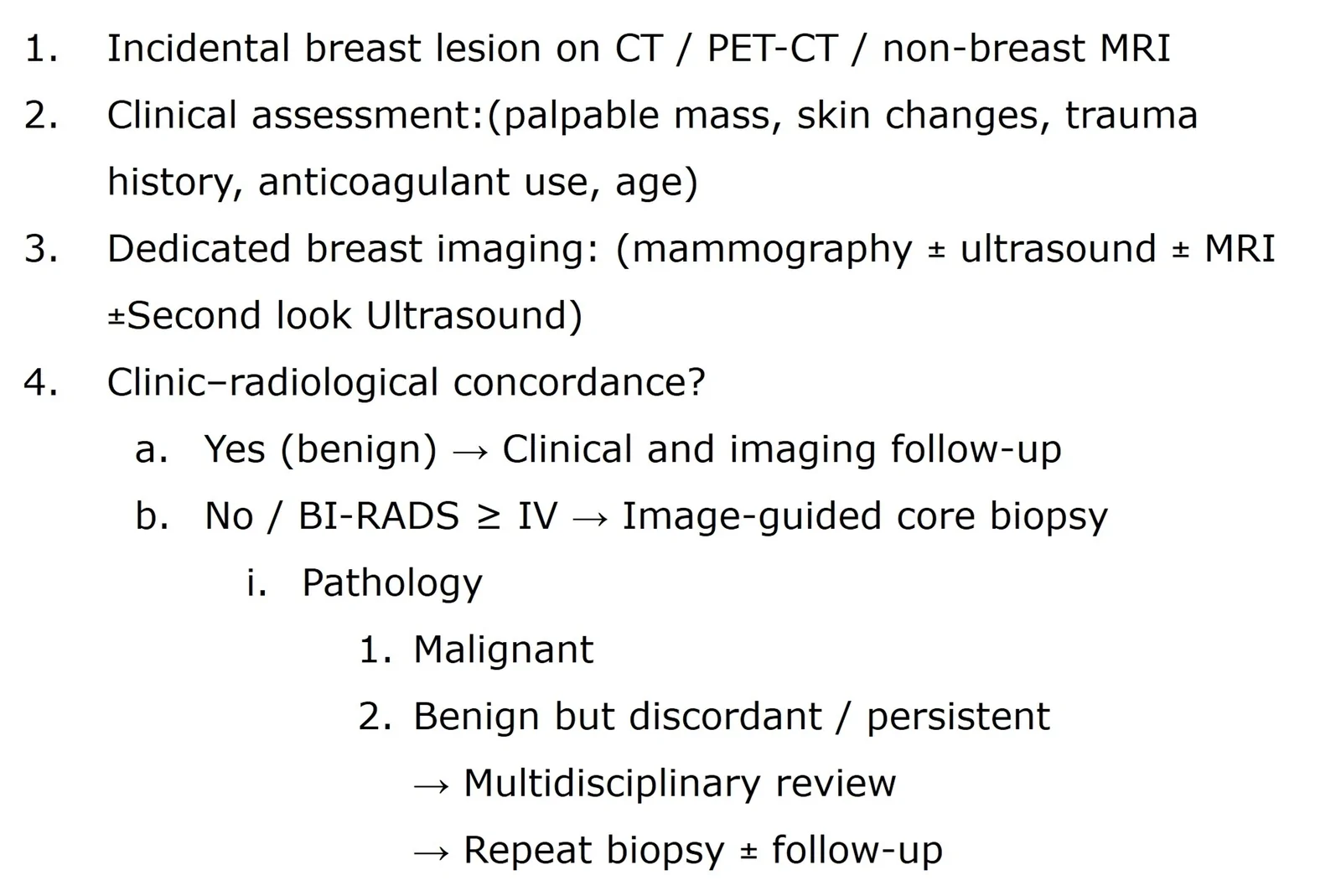
Diagnostic approach to incidental and post-traumatic breast lesions. Practical diagnostic framework for the evaluation of incidental breast findings and post-traumatic breast hematomas, created using Microsoft PowerPoint (Microsoft Corporation, Redmond, WA, USA). Patients with suspicious clinical or imaging findings undergo tissue sampling. If the pathological findings do not correlate with the clinical and imaging findings, repeat biopsy and multidisciplinary discussion are recommended.

Ultimately, this case highlights the necessity of a structured, multidisciplinary diagnostic approach to suspicious breast lesions. Integration of clinical history, physical examination, multimodal imaging, histopathology, and structured follow-up within a multidisciplinary setting is essential to balance the risk of delayed cancer diagnosis against unnecessary overtreatment. The diagnostic workflow applied in this case is summarized in Figure 11, providing a practical framework for the evaluation of traumatic and incidental breast lesions. The key principle is that when clinical or radiological doubt persists, repeated tissue sampling is mandatory to safely exclude malignancy.

## Conclusions

This case demonstrates that a post-traumatic breast hematoma can closely mimic locally advanced breast cancer, particularly in patients receiving anticoagulant therapy. Despite highly suspicious clinical and imaging findings, including a BI-RADS 4 lesion, malignancy could not be reliably confirmed or excluded by imaging alone, and even breast MRI was unable to definitively distinguish an organized hematoma from invasive breast cancer. The case highlights the importance of considering prior breast trauma in the differential diagnosis, recognizing that organized hematomas may persist for months and present with concerning clinical and radiological features. It also emphasizes the need for dedicated breast assessment of incidental findings detected on non-breast imaging and underscores the critical role of multidisciplinary evaluation, radiologic-pathologic correlation, repeat tissue sampling when necessary, and appropriate follow-up in cases of persistent clinical-radiological discordance to safely exclude malignancy.
